# Andean bears (*Tremarctos ornatus*) display selective behaviors while foraging bromeliads (*Puya spp*.) in high elevation puna grasslands

**DOI:** 10.1371/journal.pone.0314547

**Published:** 2024-12-18

**Authors:** Nicholas W. Pilfold, Denisse Mateo-Chero, William Farfan-Rios, Mrinalini Watsa, Megan A. Owen, Russell C. Van Horn

**Affiliations:** 1 Conservation Science Wildlife Health, San Diego Zoo Wildlife Alliance, Escondido, California, United States of America; 2 Department of Biology and Sabin Center for Environment and Sustainability, Wake Forest University, Winston-Salem, North Carolina, United States of America; 3 Herbario Vargas CUZ, Escuela Profesional de Biología, Universidad Nacional de San Antonio Abad del Cusco, Cusco, Peru; 4 Field Projects International, St Louis, Missouri, United States of America; Universidad Nacional de Colombia Campus Palmira, COLOMBIA

## Abstract

Andean bears (*Tremarctos ornatus*) forage extensively on bromeliads (*Puya* spp.) across their range, although their selectivity for bromeliads is less understood. We report on foraging activity by Andean bears on two species of bromeliad, *Puya leptostachya* and *Puya membranacea*, in high elevation puna grasslands (3499–3806 m.a.s.l) within and near Manu National Park (MNP) in SE Peru. We established two ridgeline transects (inside and outside MNP) with perpendicular transects running downslope. We recorded whether bromeliad plants were foraged by Andean bears on four separate sampling occasions that included wet and dry seasons from July 2017 to August 2018. We observed foraging by Andean bears at 6.8% of the available individual plants spread across 16.7% of the available patches. We utilized Resource Selection Functions to evaluate the environmental factors influencing the selection of bromeliad patches by Andean bears for foraging. Andean bears showed selection for *Puya leptostachya* over *Puya membranacea*, preferring to forage during the dry season at higher density patches of younger vegetative-stage bromeliads, possibly due to the increased bioavailability of nutrients in the basal meristematic plant tissue the bears prefer to eat. Andean bears selected bromeliad patches growing on east-facing, steep, high-altitude slopes, in a band near the cloud forest edge, which likely reflected a combination of optimal growing conditions for the bromeliads and habitat selection by the bears. Observations of foraging on grassland bromeliads occurred almost exclusively within the boundaries of MNP, which may in part reflect bears avoiding cattle impacts outside of the park. Andean bears showed active behavioral selection for bromeliads within the puna grasslands, and we recommend that grassland buffers around the cloud forest should be considered as primary habitat in conservation management plans.

## Introduction

High elevation grasslands provide numerous ecosystem services, including water and forage provisioning essential for livestock and agriculture [1, 2]. In the Andes, the conversion of native grasslands to pastures for livestock has been occurring broadly across the landscape since human arrival at least 14 thousand years ago, obscuring the true ecology and native biodiversity of unaltered grassland ecosystems [3, 4]. The human use of fire to maintain pastures has also likely contributed to limiting forest tree species from shifting their leading range edges [5], resulting in an abrupt ecotone between the cloud forest and grassland in the Andes [4, 6]. The continual elimination and fragmentation of tropical forests are likely to cause the extinction of numerous animal species [7], particularly those sensitive to disturbance [8]. For those animal species that can utilize grasslands in addition to forests, it is of critical importance to determine the suitability of the habitat, including the level of disturbance that can be tolerated, and whether the use is passive (e.g., transitions between forest patches) or active (e.g., selection of food resources).

The Andean bear (*Tremarctos ornatus*) is endemic to the Tropical Andes, found between 200 and 4,250 m.a.s.l., primarily in the cloud forest (typically 1,000–2,500 m.a.s.l.), with a species range greater than 30˚ in latitude [9, 10]. As a generalist species, Andean bears utilize a wide range of associated habitats, including tropical dry forests [11], Amazon basin forests [12], and high-elevation grasslands [13]. High-elevation grasslands in the Tropical Andes exist from the cloud forest tree line to more than 4,800 m.a.s.l. and are referred to interchangeably as the puna and páramo across the range. Habitat selection by Andean bears is often hypothesized to be linked to the utilization of food resources that have seasonal patterns of availability which the bears track [14–17]. Andean bears have an omnivorous diet, and have been observed to consume plants including the fruits and seeds, invertebrates, carrion, and vertebrates [9]. Bromeliads (Bromeliaceae), including but not limited to *Puya* spp., are usually the most frequently detected part of the Andean bear diet and therefore make up the largest component in dietary studies [18, 19]. Foraging on bromeliads is especially prominent in the puna and páramo grasslands [13–15, 20, 21], where the nutritional value of the basal meristematic tissue that the bears eat can be higher than that of bromeliads growing in the cloud forest [22].

Despite the depth of studies on Andean bear dietary composition [9], and the abundant signs the bears leave after feeding on bromeliads [23], limited evidence exists for how the bears may select for feeding locations and how preferences may be associated with bromeliad species, seasonality, and landscape. Peyton [15] observed partial bromeliad patch consumption, with bears passing over individual plants within the same patch to forage on others at a different nearby patch, suggesting a selective behavior may be involved. Small-scale studies have indicated that Andean bears consider the density of bromeliad plants when selecting patches, avoiding patches with lower densities [24, 25]. Paradoxically, the nutritional value of bromeliad plants has yet to be associated with either the patches selected for foraging [25] or the habitat Andean bears most frequently occur in [22]. Although Andean bears forage on bromeliads in the puna and páramo grasslands, it is unclear whether this is due to an active behavioral selection or only passively while traveling between preferred cloud forest habitats.

The objective of this study was to examine Andean bear foraging activity on terrestrial bromeliads in high-elevation puna grasslands for patterns consistent with an active selection of resources in the grasslands. We hypothesized that Andean bears would display behavioral selection for bromeliads in the grasslands across species, seasons, and variable landscapes. We predicted that Andean bears would prefer younger vegetative-stage bromeliads due to the higher accessibility of nutrients [26], growing in denser patches near the cloud forest edge where security from humans is highest for the bears [13]. We also predicted that Andean bears would avoid foraging in areas with higher human impacts, including the presence of livestock.

## Materials & methods

### Study area

**Fig 1:**
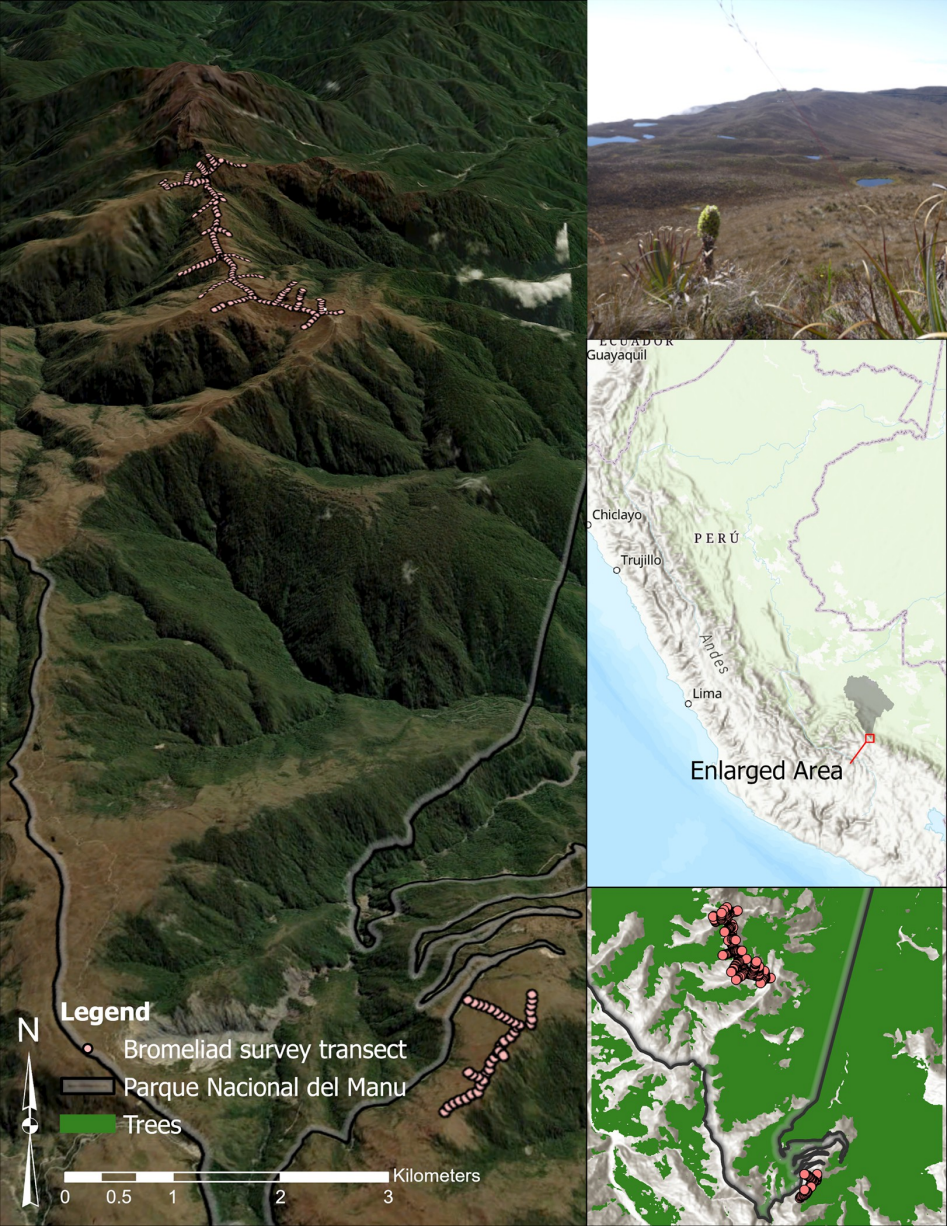
Study area in which foraging sign of terrestrial bromeliads (*Puya* spp.) in high-elevation puna grasslands by Andean bears was recorded, within and near Manu National Park, Peru. Basemaps provided by Esri [31].

Bromeliad foraging surveys were conducted in and adjacent to the UNESCO World Heritage Site of Manu National Park (MNP), Peru, geographical location coordinates 13°06’54.0"S 71°37’19.2"W (Fig 1). Surveys occurred across high-elevation moist-wet puna grasslands (3499–3806 m.a.s.l.; hereafter ‘puna grassland’), which have supported a long history of human activity [27]. Three climatic seasons can be defined: a wet season from November to March, a dry season from May to July, and an austral spring from September to October, with April and August characterized as transitional months [28]. Mean temperatures range from 4.5–8.6°C at 3600 m, with precipitation of 1500–2000 mm yr^-1^ [28]. The puna grassland in our study area was characterized by ephemeral peat bogs and waterlogged soils in glacially carved depressions [29], and drier steep slopes with stiff perennial bunch grasses [30]. Two principal species of bromeliads were patchily distributed within the puna: *Puya leptostachya* and *Puya membranacea*. *Puya membranacea* grew in denser but infrequent patches (median = 10 plants/patch), whereas *P*. *leptostachya* were more broadly distributed but in smaller patches (median = 2 plants/patch). Patches were defined as the same species of spatially contiguous bromeliads, with each individual plant growing within 1 m of its nearest neighbor plant.

### Sample collection

Observations of bromeliad foraging by Andean bears were collected on four sampling field campaigns: July 30 –August 1, 2017; October 24 –November 3, 2017; February 25 –March 10, 2018; and July 25 –August 13, 2018. Teams of three researchers walked strip transects across the puna and recorded GPS locations of bromeliads encountered. Transects were orientated both along ridgelines and downslope from ridgelines. There were two principal ridgeline transects: one 3500 m transect inside MNP and one 1000 m transect outside of MNP, with downslope transects spaced every 500 m perpendicularly from the ridgelines (Fig 1). Downslope transects continued for a maximum of 500 m from the ridgeline or until the transect reached cloud forest or was impassable on foot, whichever occurred first. Individuals within three-person teams were spaced 1 m apart, with the middle team member walking the transect line. Each team member was responsible for detecting bromeliads within a 1 m swath (0.5 m to the left and right), for a total coverage of 3 m along each strip transect to increase the detection and precision of bromeliad observations [32]. At each bromeliad patch, a central location and elevation were recorded on a handheld GPS (Garmen eTrex 30x).

**Fig 2:**
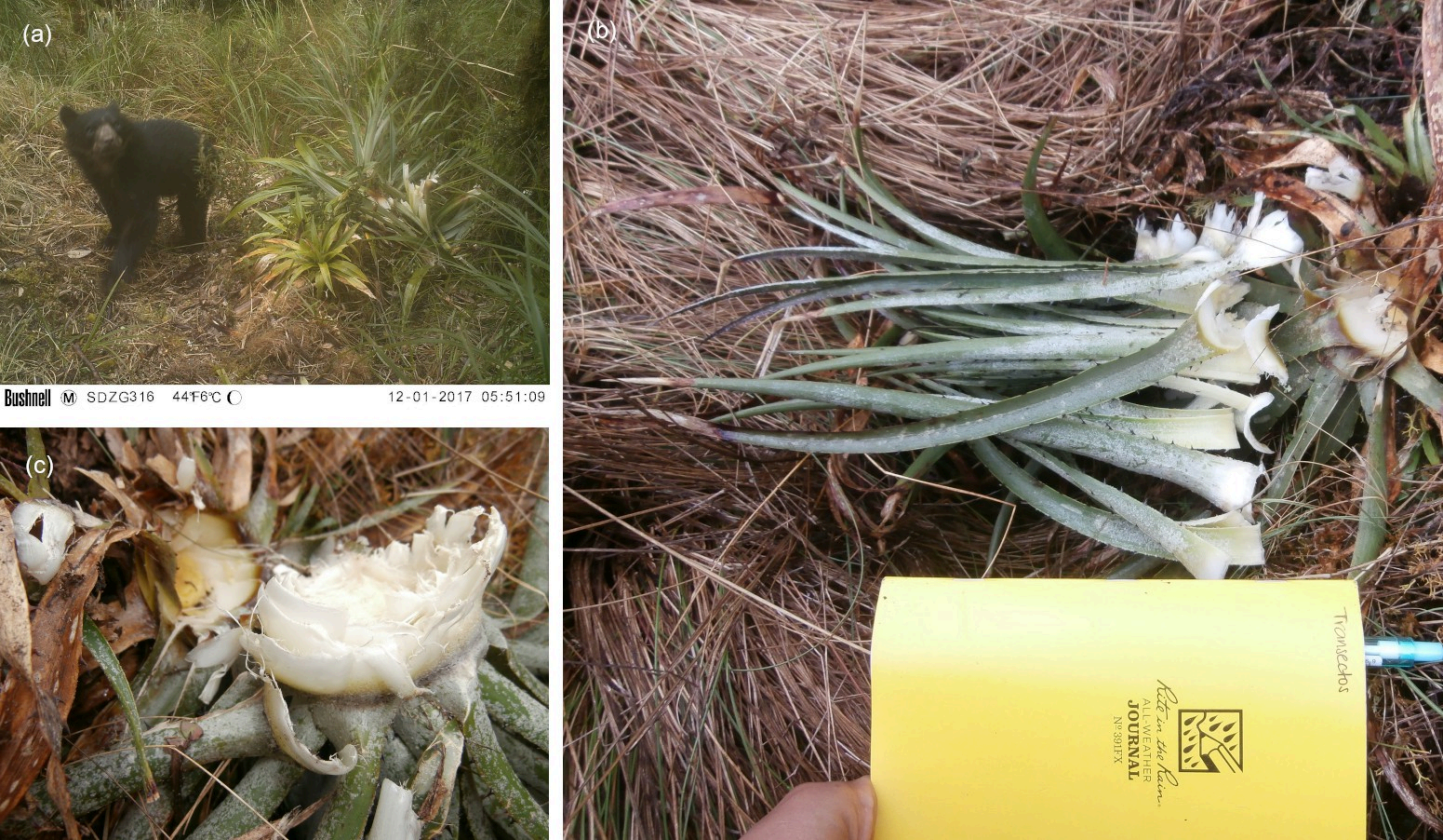
Examples of foraging sign left by Andean bears on terrestrial bromeliads in Peru. Clockwise from the top right: (a) juvenile Andean bear consuming a bromeliad at a camera station inside MNP; (b) characteristic observation of a vegetative P. leptostachya foraged by Andean bear; (c) a close up of the basal meristematic tissue that Andean bears feed on.

In addition to spatial locations of bromeliads, teams recorded the number of individual plants within a patch, species identity, and their flowering and foraged status. Species identity was confirmed by taxonomic key, and all physical specimens collected were stored at Herbario Vargas CUZ, Universidad Nacional de San Antonio Abad del Cusco. Bromeliads were categorized into four stages of the flowering process: 1) vegetative / no flowering stalk; 2) flower stalk present but no flowers; 3) active flowering; 4) post flowering seed head. Foraging status was identified from characteristic patterns of Andean bear consumption, including digging and consumption of stalk hearts (Fig 2). Confirmation of foraging by Andean bears were aided by the observations gathered by trail cameras at bromeliad patches. We deployed Bushnell Trophy Cam HD trail cameras at three sites facing bromeliad patches over the duration of our study and confirmed the presence of Andean bears near to transects during our survey period both in and outside of MNP.

### Spatial analysis

To identify the factors influencing the selection of bromeliads foraged by Andean bears, we used Resource Selection Functions (RSF). RSFs evaluated the relative likelihood of factors influencing a bear foraging at a patch of bromeliads versus those patches that were available but unused [33]. The factors influencing foraging selection was modelled using a fixed-effect exponential RSF [34]:

$$\hat{w}(x)=\exp\left({\hat{\beta }}_{1}x_{1}+{\hat{\beta }}_{2}x_{2}+\ldots +{\hat{\beta }}_{n}x_{n}\right)$$ (1)

with covariates *x_n_* and coefficients ${\hat{\beta }}_{n}$. To model habitat selection for foraging, we identified a set of covariates we hypothesized were important to Andean bears, utilizing both bromeliad surveys and remotely sensed satellite data (Table 1). Briefly, season was defined as wet or dry, with the dry season (May-October) including the austral spring prior to the start of the wet season in November. We included measures of foraging choice, including bromeliad species, patch density, and flowering status to reflect dietary need and palatability. We also examined landscape-scale habitat characteristics including the elevation, slope, and aspect of the sampling location, as well as the distance from the cloud forest edge to reflect both bromeliad growing conditions and landscape preferences of Andean bears. Finally, we included a measure of protected area status to reflect if the sampling location was inside or outside of MNP.

**Table 1:** Covariates used to model habitat selection by Andean bears while foraging bromeliads in the puna grasslands in and near to Manu National Park, Peru.

Name	Model Acronym	Data Range	Description & Source
Season	Dry	1/0	Dry season (May–October) with wet season (November–April) as ref. category (0)
Altitude	ALT + ALT^2^	3635 (3499–3806) m.a.s.l.	GPS measured meters above sea level (m.a.s.l.)
Aspect	North, East	1/0	North-facing (±45°of 0°) and east-facing (±45°of 90°) slopes [35] dummy coded as separate covariates with all other aspects as ref. category (0)
Slope	SLP	18 (0–56)°	Angle of downward sloping terrain [35]
Distance to Forest Edge	EDGE + EDGE^2^	224 (0–477) m	Distance to “Trees” as defined by ESRI 2020 Land Cover [36]
Species	P.Lep	1/0	*Puya leptostachya* with *Puya membranacea* as ref. category (0)
Density	PATCH	3 (1–179)	Number of alive plants of the same species spatially contiguous to each other (< 1m)
Flowering Status	Veg	1/0	Only vegetative stage plants in patch with patches with plants in any stage of reproductive growth as ref. category (0)
Protected Area	MNP	1/0	Inside MNP with outside MNP as ref. category (0)

We predicted that all a priori factors as represented in the table would have a positive influence on Andean bear habitat selection. Data distribution listed as mean value and range in parentheses.

### Statistical analysis

A binomial (logit link) generalized linear model (GLM) was used to maximize the use-availability likelihood and determine the top coefficients ${\hat{\beta }}_{n}$ for the RSF [37], using the package *glm* in R 4.1.2 [38]. We used Akaike Information Criterion (AIC; [39]) to select the most parsimonious model. Before model selection, we checked continuous covariates for non-linear fit using quadratic transformations, which were determined to be a better fit for distance to the forest edge (EDGE) and elevation (ALT). All continuous covariates were mean centered prior to modelling, and we examined collinearity between covariates using a Pearson’s correlation matrix [40]. We screened covariates for a correlation of r ≥ |0.6|, but no covariates showed significant lack of independence. We evaluated a balanced set of 35 a priori candidate models, with all covariates appearing equally in the candidate set so that we could define factor importance based AIC_C_ weight (*w*_*i*_). We used log-likelihood ratio tests and conditional-R2 [41] to examine top model improvement over the null. We also examined seasonal variation in the flowering status of the bromeliads and species composition using a Chi-Square test. For all statistical tests, alpha was set to 0.05.

## Results

### Bromeliad diversity & phenology

**Fig 3:**
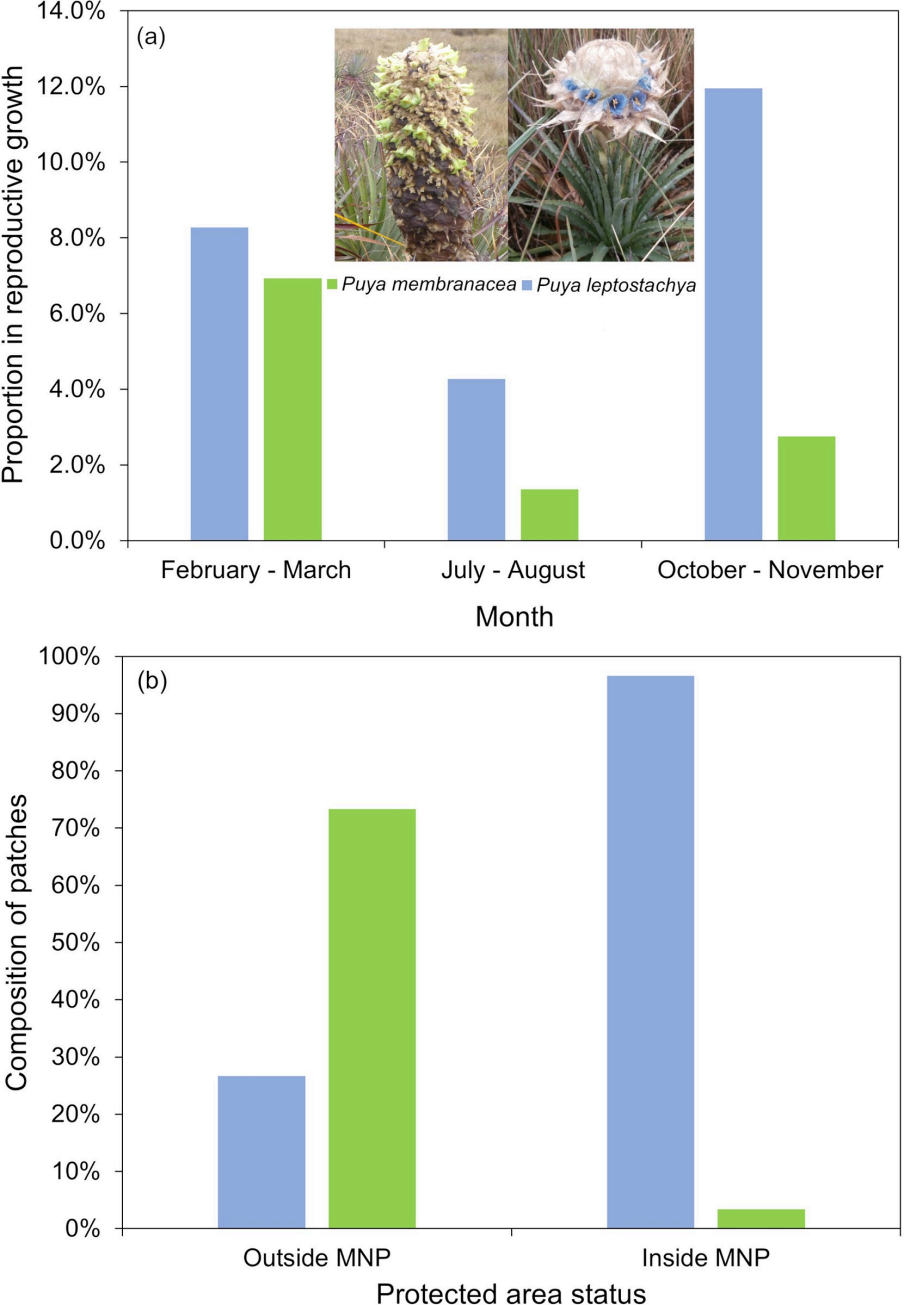
Trends in the species composition and reproductive phenology of *P*. *leptostachya* and *P*. *membranacea* within the puna grasslands of Peru. (a) proportion of plants observed in any stage of reproductive growth across season; (b) proportion of species observed inside and outside of Manu National Park.

We recorded 11,613 individual bromeliad plants across 2,895 distinct patches. Of the plants, 26.4% (3066/11613) were identified as *P*. *membranacea*, while 73.6% (8547/11613) were *P*. *leptostachya*. There was significant seasonal variation in the observation of reproductive growth for both *P*. *membranacea* (*χ*^*2*^ = 49.3, *df* = 2, *P <* 0.001) and *P*. *leptostachya* (*χ*^*2*^ = 111.7, *df* = 2, *P <* 0.001), with the lowest proportion of such observations during July-August sampling periods (Fig 3A). There was significant variation in the species composition of grassland bromeliads inside vs. outside of MNP (*χ*^*2*^ = 955, *df* = 1, *P <* 0.001), with higher proportions of *P*. *leptostachya* inside MNP (Fig 3B).

### Foraging selection by Andean bears

**Fig 4:**
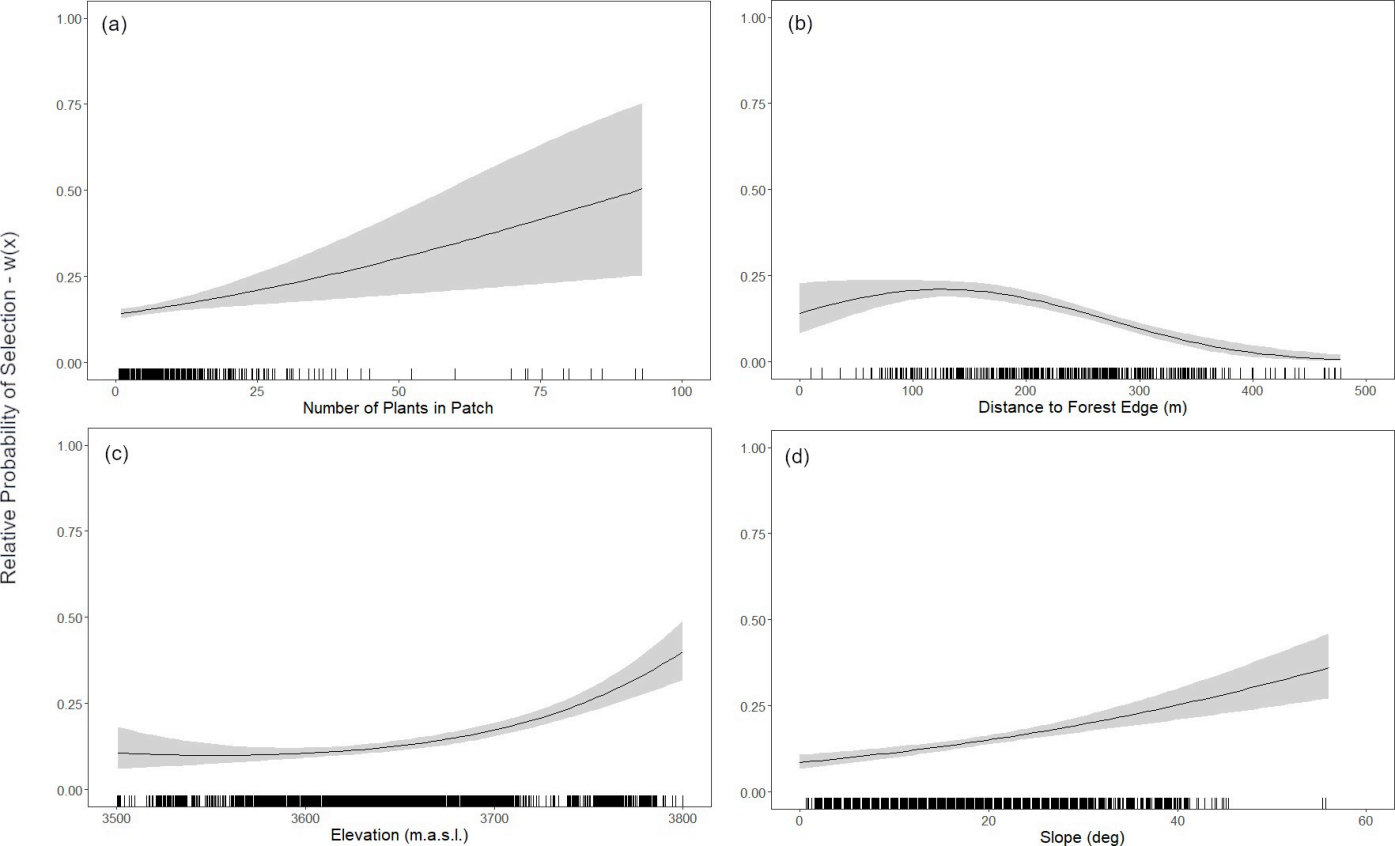
Response curves (95% CIs) from continuous covariates determined as top factors influencing the likelihood of Andean bear foraging selection for terrestrial bromeliads in puna grasslands. Top continuous factors included: (a) density of plants per patch; (b) distance to the forest edge; (c) elevation; and (d) slope. Rug plots along the bottom indicate observed sample distribution.

Observations of foraging by Andean bears occurred at 6.8% (785/11613) of the available individual plants spread across 16.7% (483/2895) of the available patches. The top RSF model for patch selection included all covariates except for north-facing aspect (AIC *w*_*i*_ = 0.33) and was a significant improvement in fit over the null model and more parsimonious than the global model (Table 2). Season was a top factor (AIC *w*_*i*_ = 1.00) in the RSF model, with Andean bears 1.79 (*CI*_*95*_ *=* 1.38–2.35) times more likely to forage for bromeliad patches in the grasslands during the dry season. Bromeliad species (AIC *w*_*i*_ = 0.98) and the patch density (AIC *w*_*i*_ = 1.00) were top factors, with Andean bears 2.71 (*CI*_*95*_ *=* 1.24–6.66) times more likely to select *P*. *leptostachya* over *P*. *membranacea*, while also selecting for patches with a greater number of available plants (Fig 4A). Flowering status (‘Veg’) was a top factor (AIC *w*_*i*_ = 1.00), with Andean bears 2.17 (*CI*_*95*_ *=* 1.51–3.21) times more likely to select patches with only vegetative stage plants than patches with plants in any stage of reproductive growth (i.e., flower stalk with buds, flowers, or seed heads). Distance to the cloud forest edge was a top factor (AIC *w*_*i*_ = 1.00), with Andean bears selecting bromeliad patches in a band closer to the forest edge (Fig 4B). Altitude (AIC *w*_*i*_ = 1.00), east-facing aspects (AIC *w*_*i*_ = 1.00), and slope (AIC *w*_*i*_ = 0.76) were all top factors in the RSF model. Andean bears were 1.47 (*CI*_*95*_ *=* 1.18–1.84) times more likely to select for bromeliad patches growing on east-facing slopes, and selected patches in higher elevations and on steeper slopes (Fig 4C and 4D). Lastly, protected area status was also a top factor (AIC *w*_*i*_ = 1.00), with Andean bears 19.43 (*CI*_*95*_ *=* 3.67–366.32) times more likely to select for bromeliad patches growing within MNP, with only a single observation of a bromeliad patch foraged outside of MNP. During our survey period, we recorded one observation of an Andean bear near to the transect outside of MNP and one observation of an Andean bear near to the transect inside MNP, confirming bear presence inside and outside of MNP.

**Table 2:** Comparison of model fit on the likelihood of selection of foraging sites by Andean bears in the puna grasslands in and near to Manu National Park, Peru.

Rank	Type	Model	*k*	*LL*	*AIC*	*ΔAIC*	*AIC w*_*i*_	*R* ^*2*^
1	Top	β_0_ + Dry + ALT + ALT^2^ + East + SLP + EDGE + EDGE^2^ + P.Lep + PATCH + Veg + MNP	12	-1060.9	2145.6	0.0	0.497	0.19
2	Global	β_0_ + Dry + ALT + ALT^2^ + North + East + SLP + EDGE + EDGE^2^ + P.Lep + PATCH + Veg + MNP	13	-258.81	2147.2	1.4	0.240	0.19
3	Null	β_0_	1	-1223.3	2448.6	303.0	0.000	

Models were fit using a binomial logit-link GLM. For the full set of a priori models, see S1 Table.

## Discussion

The Andean bear has lost much of its historic range, and its habitat is under continual threat of contraction and fragmentation due to human activity [42]. Determining the extent to which Andean bears actively utilize areas outside of their primary cloud forest habitat is a key conservation question [9, 16]. Our study, which compared the habitat use of Andean bears foraging across wet and dry seasons, inside and outside of MNP in Peru, adds evidence to the importance of puna grasslands as a key habitat for Andean bears [17, 43, 44]. We determined that Andean bears displayed seasonal, habitat, and plant selection patterns when they foraged on terrestrial bromeliad patches within the puna grasslands, consistent with an active behavioral choice for resources rather than opportunistic encounters. Andean bears strongly avoided areas with current pastoral activities when selecting foraging patches, but also selected for areas with long-standing historic anthropogenic modifications of the grasslands.

Results suggested that active livestock disturbance reduced Andean bear foraging activity in the puna grasslands, as bears in our study foraged on terrestrial bromeliads almost exclusively within MNP. This may be due to a direct avoidance of cattle presence outside of MNP, and/or the impacts of the cattle on the relative composition of terrestrial bromeliads within the puna grasslands. Andean bears preferred to forage on *P*. *leptostachya* in the dry season, which is the same time of year that livestock are moved rotationally into upland areas [45]. Rojas-VeraPinto, Bautista [46] found that in this same landscape, the closer Andean bears were to pastures and agricultural land, the higher the likelihood of conflict with people. Additionally, Melo-Dias, Huatuco [17] found that despite the importance of *Puya* spp in grasslands to Andean bear occupancy, cattle presence displaced the bears. Our results are concordant with the hypothesis that Andean bears will actively avoid livestock in the dry season [47], and that the avoidance of human disturbance has dietary implications [48]. Livestock can also alter the composition of the plant community in high-elevation grasslands through grazing and trampling of vegetation [45, 49]. Our results showed a significant decline in the proportion of available *P*. *leptostachya* patches outside of MNP. Because *P*. *leptostachya* are physically smaller and grow in lower density patches broadly distributed within the grassland, they may be more exposed to the grazing impacts of cattle than *P*. *membranacea*. Andean bears preferred to forage on *P*. *leptostachya* over *P*. *membranacea* and the reduction in the availability of *P*. *leptostachya* outside of MNP may have contributed to the observed decline in foraging activity, including during periods when cattle were not present.

While we detected an effect on foraging activity related to active livestock disturbance, Andean bears selected for puna grassland areas within MNP that have had thousands of years of historic human influence [27]. The puna grasslands that Andean bears selected for foraging bromeliads have only been protected from livestock impacts since MNP was established in 1973. There are still remnants of livestock fencing within the puna grasslands inside MNP (Pilfold, pers. obs.), suggesting a relatively recent cessation of livestock keeping. Given that Andean bears are actively selecting for resources within MNP puna grasslands a relatively short duration after the disturbance ended, is suggestive of a behavioral flexibility to respond quickly once the immediate disturbance pressure is removed. Thus, the reduction or removal of cattle grazing from puna grasslands may quickly increase the amount of seasonal foraging habitat for Andean bears.

A long-standing but little tested hypothesis is that Andean bears select habitats seasonally based on the availability of food resources [9, 14–16]. Consistent with this hypothesis, Andean bears preferred the dry season to forage in the puna grasslands, selecting for higher-density patches of vegetative-stage bromeliads. Seasonal selection for grassland bromeliads supports the findings of a broadening dietary niche breadth during the dry season [19], and the selection for patches with higher densities of plants is consistent with previous findings [24, 25]. The seasonal preference also coincided with the lowest occurrence of reproductive growth (flowering) in both species of bromeliads, suggesting a possible nutritional mechanism [22]. We found that Andean bears selected patches of younger vegetative-stage bromeliads that did not contain plants in any stage of reproductive growth. Because Andean bears have short gastrointestinal tracts, which is characteristic of carnivores, they are likely to consider the digestibility and palatability of bromeliads while foraging [26]. Similar to other bear species [50, 51], fibrous content in the tissue of plants is likely an impediment to the bioavailability of proteins, fats, minerals, and sugars for Andean bears, possibly driving their preference to forage on younger and more tender plant structures that are not yet fully lignified.

Andean bears selected for high-elevation, east-facing, steep slopes in a band near to the cloud forest while foraging on patches of bromeliads. Bears selected for steeper slopes when foraging terrestrial bromeliads, but within the limits (< 60°) established in other studies [52]. The use of high-elevation areas to forage on bromeliads in the grasslands close to the cloud forest is consistent with previous studies [13, 25, 43]. Terrestrial *Puya* spp. are known to inhabit high-elevation areas characterized by extreme climatic conditions with high solar radiation [53]. The relative probability of foraging peaked at 130 m from the cloud forest edge. Closer to the forest, shade from trees may lower solar radiation for terrestrial bromeliads and reduce forage quality. Selection for foraging patches declined to near zero relative probability beyond 400 m from the forest, which may be related to the use of the cloud forest by bears as protection and cover [13].

## Conclusion

The lack of available high-quality habitat is often cited as the primary threat to Andean bears [9, 42]. However, limited information exists on how Andean bears select for resources, hampering the quantitative identification of high-quality habitat. Using one of the largest samples of foraging sign collected to date, we showed how Andean bears adjust their foraging selection of bromeliad patches across season, habitat, and plant characteristics in the puna grasslands of Peru. We suggest that the puna grasslands are a key habitat utilized by Andean bears, especially the ecotone near to the cloud forest edge where the bears preferred to forage. We recommend that future studies examine the nutritional composition of bromeliads as it relates to plant phenology and bear energetics, as well understand how diet and movement patterns of Andean bears are linked. We also recommend that conservation management plans consider the role of livestock to the sustainability of puna grasslands, and their impact on the diversity of plant species of importance to endemic wildlife.

## Supporting information

S1 Table A priori model set of variables used to model habitat selection by Andean bears foraging bromeliads in high-elevation puna grasslands, Peru. Models were fit using a binomial (logit link) generalized linear model (GLM) and Akaike’s information criterion for small samples (AICC) to rank model performance. The a priori model set is balanced so each variable is equally represented, and measures of variable importance are defined by the relative AICC weight.
(DOCX)

S1 File Andean bear bromeliad foraging dataset in high-elevation puna grasslands, Peru. (XLSX)

## References

[1] Mosquera GM, Marín F, Stern M, Bonnesoeur V, Ochoa-Tocachi BF, Román-Dañobeytia F, et al. Progress in understanding the hydrology of high-elevation Andean grasslands under changing land use. Science of The Total Environment. 2022;804:150112. doi: 10.1016/j.scitotenv.2021.150112 34520909

[2] Jäger H, Peratoner G, Tappeiner U, Tasser E. Grassland biomass balance in the European Alps: current and future ecosystem service perspectives. Ecosystem Services. 2020;45:101163. doi: 10.1016/j.ecoser.2020.101163

[3] Sylvester SP, Heitkamp F, Sylvester MDPV, Jungkunst HF, Sipman HJM, Toivonen JM, et al. Relict high-Andean ecosystems challenge our concepts of naturalness and human impact. Scientific Reports. 2017;7(1):3334. doi: 10.1038/s41598-017-03500-7 28611464 PMC5469861

[4] Bush MB, Rozas-Davila A, Raczka M, Nascimento M, Valencia B, Sales RK, et al. A palaeoecological perspective on the transformation of the tropical Andes by early human activity. Philosophical Transactions of the Royal Society B: Biological Sciences. 2022;377(1849):20200497. doi: 10.1098/rstb.2020.0497 35249394 PMC8899620

[5] Di Pasquale G, Marziano M, Impagliazzo S, Lubritto C, De Natale A, Bader MY. The Holocene treeline in the northern Andes (Ecuador): First evidence from soil charcoal. Palaeogeography, Palaeoclimatology, Palaeoecology. 2008;259(1):17–34. doi: 10.1016/j.palaeo.2006.12.016

[6] Rehm EM, Feeley KJ. The inability of tropical cloud forest species to invade grasslands above treeline during climate change: potential explanations and consequences. Ecography. 2015;38(12):1167–75. doi: 10.1111/ecog.01050

[7] Alroy J. Effects of habitat disturbance on tropical forest biodiversity. Proceedings of the National Academy of Sciences. 2017;114(23):6056–61. doi: 10.1073/pnas.1611855114 28461482 PMC5468684

[8] Betts MG, Wolf C, Pfeifer M, Banks-Leite C, Arroyo-Rodríguez V, Ribeiro DB, et al. Extinction filters mediate the global effects of habitat fragmentation on animals. Science. 2019;366(6470):1236–9. doi: 10.1126/science.aax9387 31806811

[9] Garcia-Rangel S. Andean bear Tremarctos ornatus natural history and conservation. Mammal Review. 2012;42(2):85–119. doi: 10.1111/j.1365-2907.2011.00207.x

[10] Vela-Vargas IM, Jorgenson JP, González-Maya JF, Koprowski JL. Tremarctos ornatus (Carnivora: Ursidae). Mammalian Species. 2021;53(1006):78–94.

[11] Appleton RD, Van Horn RC, Noyce KV, Spady TJ, Swaisgood RR, Arcese P. Phenotypic plasticity in the timing of reproduction in Andean bears. Journal of Zoology. 2018;305(3):196–202. doi: 10.1111/jzo.12553

[12] Figueroa J. Presencia del oso andino Tremarctos ornatus (Carnivora: Ursidae) en el bosque tropical amazónico del Peru. Acta Zoológica Mexicana. 2012;28:594–606.

[13] Peyton B, editor Habitat components of the spectacled bear in Machu Picchu, Peru. Bears—Thier Biology and Management Seventh International Conference on Bear Research and Management; 1987; Williamsburg, Virginia, USA and Plitvice Lakes, Yugoslavia.

[14] Figueroa J. Composición de la dieta del oso andino Tremarctos ornatus (Carnivora: Ursidae) en nueve áreas naturales protegidas del Perú. Therya. 2013;4(2):327–59.

[15] Peyton B., Ecology distribution, and food habits of Spectacled bears, *Tremarctos ornatus*, in Peru. Journal of Mammology. 1980;61(4):639–52. doi: 10.2307/1380309

[16] Morrell N, Appleton RD, Arcese P. Roads, forest cover, and topography as factors affecting the occurrence of large carnivores: The case of the Andean bear (Tremarctos ornatus). Global Ecology and Conservation. 2021;26. doi: 10.1016/j.gecco.2021.e01473

[17] Melo-Dias M, Huatuco JFA, Arizapana-Almonacid MA, Castañeda-Tinco MI, Chanamé F, Passamani M. Lighting up mountain coexistence: Understanding the effects of environment and livestock on habitat use by Andean bear in a conflict zone in Peruvian Andes. Journal for Nature Conservation. 2024;81:126677. doi: 10.1016/j.jnc.2024.126677

[18] Suarez L. Seasonal distribution and food habits of Spectacled Bears *Tremarctos ornatu*s in the highlands of Ecuador. Studies on Neotropical Fauna and Environment. 1988;23(3):133–6. doi: 10.1080/01650528809360755

[19] Cáceres-Martínez CH, Sánchez Montano LR, Acevedo AA, González-Maya JF. Diet of Andean bears in Tamá National Natural Park, Colombia. Ursus. 2020;2020(31e10):1–11. doi: 10.2192/URSUS-D-18-00006.1

[20] Chávez AM, Díaz C, Amanzo JM. Seasonality of Andean Bear Scat Contents in Amazonas, Northeastern Peru. Ursus. 2021;2021(32e17). doi: 10.2192/ursus-d-20-00011.2

[21] Rivadeneira-Canedo C. Estudio del oso andino (Tremarctos ornatus) como dispersor legítimo de semillas y elementos de su dieta en la región de Apolobamba-Bolivia. Ecología en Bolivia. 2008;43(1):29–40.

[22] Bernátková A, Pařiková A, Cisneros R, Čupić S, Ceacero F. Ecological effects on the nutritional value of bromeliads, and its influence on Andean bears’ diet selection. Ursus. 2021;2021(32e21):1–8. doi: 10.2192/URSUS-D-20-00021.2

[23] Goldstein IR. Andean Bear Use of the epiphytic bromeliad *Tillandsia fendleri* at Quebrada el Molino, Venezuela. Ursus. 2004;15(1):54–6.

[24] Goldstein IR, Salas L. Foraging pattern on *Puya* sp. (Bromeliacae) by *Tremarctos ornatus* (Ursidae) at Paramo El Tambor, Venezuela. Ecotropicos. 1993;6(2):24–9.

[25] DeMay SM, Roon DA, Rachlow JL, Cisneros R. Selective foraging on bromeliads by Andean bears in the Ecuadorian páramo. Ursus. 2014;25(2):139–47. doi: 10.2192/ursus-d-14-00022.1

[26] Goldman I, Silver SC, Dierenfeld ES. Passage and digestion in the spectacled bear (*Tremarctos ornatus*) fed a zoo-based diet moderately high in fiber. Proceedings of the Nutrition Advisory Group. 2001;4:92.

[27] Becerra JAB, Bitencourt MD. Ecological zoning of an Andean grasslands (puna) at the manu biosphere reserve, Peru. International Journal of Environment and Sustainable Development. 2007;6(4):357–72. doi: 10.1504/IJESD.2007.016240

[28] Rapp JM, Silman MR. Diurnal, seasonal, and altitudinal trends in microclimate across a tropical montane cloud forest. Climate Research. 2012;55(1):17–32. doi: 10.3354/cr01127

[29] Kuentz A, de Mera AG, Ledru M-P, Thouret J-C. Phytogeographical data and modern pollen rain of the puna belt in southern Peru (Nevado Coropuna, Western Cordillera). Journal of Biogeography. 2007;34(10):1762–76. doi: 10.1111/j.1365-2699.2007.01728.x

[30] Thomas B, Winterhalder B. Physical and biotic environment of southern highland Peru. In: Baker P, Little M, editors. Man in the Andes: A Multidisciplinary Study of High-Altitude Quechua 1976.

[31] Esri. "World Imagery" [basemap]. April 2024 ed. https://www.arcgis.com/home/item.html?id=10df2279f9684e4a9f6a7f08febac2a9: ArcGIS Pro 3.1; 2024.

[32] Buckland ST, Borchers DL, Johnston A, Henrys PA, Marques TA. Line transect methods for plant surveys. Biometrics. 2007;63(4):989–98. doi: 10.1111/j.1541-0420.2007.00798.x 18078477

[33] Boyce MS, McDonald LL. Relating populations to habitats using resource selection functions. Trends in Ecology & Evolution. 1999;14(7):268–72. doi: 10.1016/s0169-5347(99)01593-1 10370262

[34] Manly BFJ. Estimating a resource selection function with line transect sampling. Journal of Applied Mathematics and Decision Sciences. 2002;6(4):213–28. doi: 10.1207/S15327612JAMD0604_3

[35] Esri. "Terrain" [basemap]. November 2023 ed. https://www.arcgis.com/home/item.html?id=58a541efc59545e6b7137f961d7de883: ArcGIS Pro 3.1; 2023.

[36] Karra K, Kontgis C, Statman-Weil Z, Mazzariello JC, Mathis M, Brumby SP, editors. Global land use / land cover with Sentinel 2 and deep learning. 2021 IEEE International Geoscience and Remote Sensing Symposium IGARSS; 2021 11–16 July 2021.

[37] McDonald TL. The point process use-availability or presence-only likelihood and comments on analysis. Journal of Animal Ecology. 2013;82(6):1174–82. doi: 10.1111/1365-2656.12132 24111555

[38] R Core Team. R: A Language and Environment for Statistical Computing. Vienna, Austria: R Foundation for Statistical Computing; 2023.

[39] Burnham KP, Anderson DR. Model Selection and Multimodel Inference: A Practical Information-Theoretic Approach. 2 ed. New York, NY: Springer; 2002.

[40] DeLong ER, DeLong DM, Clarke-Pearson DL. Comparing the Areas under Two or More Correlated Receiver Operating Characteristic Curves: A Nonparametric Approach. Biometrics. 1988;44(3):837–45. doi: 10.2307/2531595 3203132

[41] Nakagawa S, Schielzeth H. A general and simple method for obtaining R2 from generalized linear mixed-effects models. Methods in Ecology and Evolution. 2013;4(2):133–42. doi: 10.1111/j.2041-210x.2012.00261.x

[42] Velez-Liendo X, García-Rangel S. *Tremarctos ornatus* (errata version published in 2018). e.T22066A123792952: The IUCN Red List of Threatened Species 2017; 2017.

[43] Rodríguez D, Reyes A, Tarquino-Carbonel AdP, Restrepo H, Reyes-Amaya N. Space use by a male Andean bear (*Tremarctos ornatus*) tracked with GPS telemetry in the Macizo Chingaza, Cordillera Oriental of the Colombian Andes. Notas sobre Mamíferos Sudamericanos. 2021;03(1):001–8. doi: 10.31687/saremNMS.21.2.4

[44] Peralvo MF, Cuesta F, van Manen F. Delineating priority habitat areas for the conservation of Andean bears in northern Ecuador. Ursus. 2005;16(2):222–33. doi: 10.2192/1537-6176(2005)016[0222:DPHAFT]2.0.CO;2

[45] Becerra JAB. Grazing Intensity, Plant Diversity, and Rangeland Conditions in the Southeastern Andes of Peru (Palccoyo, Cusco). Land Use Change and Mountain Biodiversity: CRC Press; 2006. p. 153–66.

[46] Rojas-VeraPinto R, Bautista C, Selva N. Living high and at risk: predicting Andean bear occurrence and conflicts with humans in southeastern Peru. Global Ecology and Conservation. 2022;36. doi: 10.1016/j.gecco.2022.e02112

[47] Aurich-Rodriguez F, Piana RP, Appleton RD, Burton AC. Threatened Andean bears are negatively affected by human disturbance and free-ranging cattle in a protected area in northwest Peru. Mammalian Biology. 2022;102(1):177–87. doi: 10.1007/s42991-021-00217-z

[48] Hernani-Lineros L, Garcia E, Pacheco LF. Andean bear diet near to and far from a road. Ursus. 2020;2020(31e7):1–7. doi: 10.2192/URSUS-D-19-0003.1

[49] Sala O, Oesterheld M, León R, Soriano A. Grazing effects upon plant community structure in subhumid grasslands of Argentina. Vegetatio. 1986;67:27–32.

[50] Christian AL, Knott KK, Vance CK, Falcone JF, Bauer LL, Fahey GC Jr, et al. Nutrient and mineral composition during shoot growth in seven species of Phyllostachys and Pseudosasa bamboo consumed by giant panda. Journal of Animal Physiology and Animal Nutrition. 2015;99(6):1172–83. doi: 10.1111/jpn.12287 25581029

[51] Rode KD, Robbins CT, Shipley LA. Constraints on herbivory by grizzly bears. Oecologia. 2001;128(1):62–71. doi: 10.1007/s004420100637 28547091

[52] Cuesta F, Peralvo MF, van Manen FT. Andean Bear Habitat Use in the Oyacachi River Basin, Ecuador. Ursus. 2003;14(2):198–209.

[53] Krömer T, Kessler M, Herzog SK. Distribution and flowering ecology of bromeliads along two climatically contrasting elevational transects in the Bolivian Andes. Biotropica. 2006;38(2):183–95. doi: 10.1111/j.1744-7429.2006.00124.x

