# Supplementary material for: Andean bears (Tremarctos ornatus) display selective behaviors while foraging bromeliads (Puya spp.) in high elevation puna grasslands
Source: PLoS One. 2024 Dec 18;19(12):e0314547. doi: 10.1371/journal.pone.0314547 (PMC11654964; doi:10.1371/journal.pone.0314547)
Supplement: S1 Table — Models were fit using a binomial (logit link) generalized linear model (GLM) and Akaike’s information criterion for small samples (AICC) to rank model performance. The a priori model set is balanced so each variable is equally represented, and measures of variable importance are defined by the relative AICC weight. (DOCX) [file pone.0314547.s001.docx]

**Supporting Information S1 Table**

Title

Andean bears (*Tremarctos ornatus*) display selective behaviors while foraging bromeliads (*Puya spp.*) in high elevation puna grasslands

Authors

Nicholas W. Pilfold^1^*, Denisse Mateo-Chero^1^, William Farfan-Rios ^2,3^, Mrinalini Watsa^1,4^, Megan A. Owen^1^, Russell C. Van Horn^1^

*Corresponding Author

Institutional Addresses

^1^Conservation Science & Wildlife Health, San Diego Zoo Wildlife Alliance, Escondido, 92027, United States

^2^ Department of Biology and Sabin Center for Environment and Sustainability, Wake Forest University, Winston-Salem, NC, USA

^3^ Herbario Vargas CUZ, Escuela Profesional de Biología, Universidad Nacional de San Antonio Abad del Cusco, Cusco, Peru

^4^ Field Projects International, St Louis, United States

**Table S1** A priori model set of variables used to model habitat selection by Andean bears foraging bromeliads in high-elevation puna grasslands, Peru. Models were fit using a binomial (logit link) generalized linear model (GLM) and Akaike’s information criterion for small samples (AIC_C_) to rank model performance. The a priori model set is balanced so each variable is equally represented, and measures of variable importance are defined by the relative AIC_C_ weight.

| Model Rank | Dry | ALT + ALT^2^ | P.Lep | PATCH | Veg | EDGE + EDGE^2^ | SLP | North | East | MNP | *k* | *AIC_C_* | *∆ AIC_C_* | *w_i_* |
| --- | --- | --- | --- | --- | --- | --- | --- | --- | --- | --- | --- | --- | --- | --- |
| 1 | 1 | 1 | 1 | 1 | 1 | 1 | 1 | 0 | 1 | 1 | 12 | 2145.781 | 0.000 | 0.497 |
| 2 | 1 | 1 | 1 | 1 | 1 | 1 | 1 | 1 | 1 | 1 | 13 | 2147.236 | 1.455 | 0.240 |
| 3 | 1 | 1 | 1 | 1 | 1 | 1 | 0 | 0 | 1 | 1 | 11 | 2147.926 | 2.145 | 0.170 |
| 4 | 1 | 1 | 1 | 1 | 1 | 1 | 0 | 1 | 1 | 1 | 12 | 2149.727 | 3.946 | 0.069 |
| 5 | 1 | 1 | 0 | 1 | 1 | 1 | 1 | 1 | 1 | 1 | 12 | 2152.177 | 6.396 | 0.020 |
| 6 | 1 | 1 | 1 | 1 | 1 | 1 | 1 | 1 | 0 | 1 | 12 | 2155.891 | 10.110 | 0.003 |
| 7 | 1 | 1 | 1 | 1 | 1 | 1 | 1 | 1 | 1 | 0 | 12 | 2162.075 | 16.294 | 0.000 |
| 8 | 0 | 1 | 1 | 1 | 1 | 1 | 1 | 1 | 1 | 1 | 12 | 2163.755 | 17.974 | 0.000 |
| 9 | 1 | 1 | 1 | 1 | 1 | 1 | 1 | 1 | 0 | 0 | 11 | 2169.319 | 23.538 | 0.000 |
| 10 | 1 | 1 | 0 | 0 | 1 | 1 | 1 | 1 | 1 | 1 | 11 | 2170.809 | 25.028 | 0.000 |
| 11 | 1 | 1 | 1 | 0 | 1 | 1 | 1 | 1 | 1 | 1 | 12 | 2172.496 | 26.715 | 0.000 |
| 12 | 1 | 1 | 1 | 1 | 0 | 1 | 1 | 1 | 1 | 1 | 12 | 2176.963 | 31.182 | 0.000 |
| 13 | 1 | 1 | 1 | 1 | 0 | 1 | 1 | 0 | 0 | 1 | 10 | 2183.503 | 37.722 | 0.000 |
| 14 | 0 | 1 | 0 | 0 | 1 | 1 | 1 | 1 | 1 | 1 | 10 | 2190.766 | 44.985 | 0.000 |
| 15 | 0 | 1 | 0 | 1 | 0 | 1 | 1 | 0 | 1 | 1 | 9 | 2197.196 | 51.415 | 0.000 |
| 16 | 1 | 0 | 1 | 1 | 1 | 1 | 1 | 1 | 1 | 1 | 11 | 2214.504 | 68.723 | 0.000 |
| 17 | 1 | 0 | 1 | 1 | 1 | 1 | 0 | 0 | 0 | 1 | 8 | 2234.758 | 88.977 | 0.000 |
| 18 | 0 | 0 | 1 | 1 | 1 | 1 | 1 | 1 | 1 | 1 | 10 | 2238.409 | 92.628 | 0.000 |
| 19 | 0 | 1 | 0 | 0 | 0 | 1 | 1 | 0 | 0 | 0 | 6 | 2245.322 | 99.541 | 0.000 |
| 20 | 1 | 0 | 1 | 0 | 1 | 1 | 1 | 1 | 0 | 1 | 9 | 2248.848 | 103.067 | 0.000 |
| 21 | 1 | 1 | 1 | 1 | 0 | 0 | 1 | 1 | 1 | 1 | 10 | 2284.209 | 138.428 | 0.000 |
| 22 | 1 | 1 | 1 | 1 | 1 | 0 | 1 | 1 | 1 | 1 | 11 | 2284.209 | 138.428 | 0.000 |
| 23 | 1 | 0 | 1 | 1 | 1 | 0 | 1 | 1 | 1 | 0 | 8 | 2292.774 | 146.993 | 0.000 |
| 24 | 0 | 0 | 1 | 1 | 0 | 1 | 0 | 0 | 1 | 1 | 7 | 2295.031 | 149.250 | 0.000 |
| 25 | 0 | 0 | 1 | 1 | 1 | 1 | 0 | 0 | 0 | 1 | 7 | 2297.750 | 151.969 | 0.000 |
| 26 | 0 | 1 | 1 | 1 | 1 | 0 | 0 | 1 | 1 | 0 | 8 | 2297.959 | 152.178 | 0.000 |
| 27 | 1 | 0 | 0 | 1 | 0 | 1 | 0 | 0 | 0 | 0 | 5 | 2337.427 | 191.646 | 0.000 |
| 28 | 1 | 1 | 0 | 0 | 0 | 0 | 1 | 1 | 1 | 0 | 7 | 2338.144 | 192.363 | 0.000 |
| 29 | 0 | 1 | 0 | 0 | 1 | 0 | 0 | 0 | 0 | 0 | 4 | 2338.668 | 192.887 | 0.000 |
| 30 | 1 | 0 | 0 | 1 | 1 | 0 | 1 | 1 | 0 | 0 | 6 | 2340.821 | 195.040 | 0.000 |
| 31 | 1 | 1 | 1 | 0 | 0 | 0 | 0 | 0 | 1 | 1 | 7 | 2341.306 | 195.525 | 0.000 |
| 32 | 1 | 0 | 0 | 0 | 1 | 0 | 0 | 1 | 0 | 0 | 4 | 2363.608 | 217.827 | 0.000 |
| 33 | 0 | 1 | 0 | 0 | 0 | 0 | 0 | 1 | 1 | 0 | 5 | 2377.932 | 232.151 | 0.000 |
| 34 | 0 | 0 | 0 | 0 | 0 | 0 | 1 | 1 | 1 | 0 | 4 | 2417.450 | 271.669 | 0.000 |
| 35 | 0 | 0 | 1 | 0 | 0 | 0 | 0 | 0 | 0 | 1 | 3 | 2420.431 | 274.650 | 0.000 |
| Total | 23 | 23 | 23 | 23 | 23 | 23 | 23 | 23 | 23 | 23 |  |  |  |  |
